# Cyber Surveillance for Flood Disasters

**DOI:** 10.3390/s150202369

**Published:** 2015-01-22

**Authors:** Shi-Wei Lo, Jyh-Horng Wu, Fang-Pang Lin, Ching-Han Hsu

**Affiliations:** 1 National Center for High-Performance Computing, No. 7, R&D 6th Rd., Hsinchu Science Park, Hsinchu City 30076, Taiwan; E-Mails: LSW@nchc.narl.org.tw (S.-W.L.); jhwu@nchc.narl.org.tw (J.-H.W.); fplin@nchc.narl.org.tw (F.-P.L.); 2 Department of Biomedical Engineering and Environmental Sciences, National Tsing Hua University, No. 101, Section 2, Kuang-Fu Road, Hsinchu 30013, Taiwan

**Keywords:** flood, first warning, early warning system, image segmentation, video surveillance

## Abstract

Regional heavy rainfall is usually caused by the influence of extreme weather conditions. Instant heavy rainfall often results in the flooding of rivers and the neighboring low-lying areas, which is responsible for a large number of casualties and considerable property loss. The existing precipitation forecast systems mostly focus on the analysis and forecast of large-scale areas but do not provide precise instant automatic monitoring and alert feedback for individual river areas and sections. Therefore, in this paper, we propose an easy method to automatically monitor the flood object of a specific area, based on the currently widely used remote cyber surveillance systems and image processing methods, in order to obtain instant flooding and waterlogging event feedback. The intrusion detection mode of these surveillance systems is used in this study, wherein a flood is considered a possible invasion object. Through the detection and verification of flood objects, automatic flood risk-level monitoring of specific individual river segments, as well as the automatic urban inundation detection, has become possible. The proposed method can better meet the practical needs of disaster prevention than the method of large-area forecasting. It also has several other advantages, such as flexibility in location selection, no requirement of a standard water-level ruler, and a relatively large field of view, when compared with the traditional water-level measurements using video screens. The results can offer prompt reference for appropriate disaster warning actions in small areas, making them more accurate and effective.

## 1. Introduction

Intense rainfall within a short period can be caused by extreme weather conditions as a result of climate change. When a large amount of water cannot be drained in time within the rainfall area, we face river overflow or urban inundation, which frequently causes a large number of casualties and a considerable property loss. Therefore, effective near real-time hydrological information is extremely important for flood warning and advance resident evacuation. Recently, there have been several studies on both forecasting- and monitoring-based flooding warning. At present, the major sources of the forecast estimation are large-scale remote sensing methods, including meteorology satellites and radar-based quantitative precipitation estimation. The forecast estimation utilizing multiple forecast models, the ensemble forecast technique can be used for performing simulations with integrated remote and on-site observation data; the analysis results can be further used for forecasting future precipitation or flooding information [1–5]. However, as a result of the complex interactions in the atmosphere, the accuracy of precipitation forecasting is still a key issue in this field. At the same time, the spatial resolution of a rainfall simulation is limited by numerous computing needs. Therefore, it is still very difficult to perform precise precipitation forecasting in small-scale areas. On the other hand, the flood monitoring is using on-site water-level measurement facilities, such as rainfall observation stations, water level observation stations, and meteorological stations. These on-site stations can directly measure the water or rainfall levels and provide instant notifications. However, direct sensor measurement of the water level is restricted by the particular limitations of the sensor installation location and the unavoidable requirement of frequent maintenance. It also has the disadvantage of obtaining only water-level information and not visual evidence for judgment. Therefore, recently, the integration of flood monitoring systems and image processing techniques for flooding and inundation monitoring has become vital for flood disaster prevention. In this paper, we propose a visual flood monitoring system for near real-time flood overflow detection and flood risk evaluation using remote surveillance videos. The proposed system can be used as a cyber surveillance tool for instant flood monitoring and warning.

Today, all the developed countries in the world are using a variety of weather forecasting systems to assist disaster prevention, relief, and evacuation, in order to drastically reduce the number of casualties and the amount of economic loss caused by disastrous weather conditions [4,6–9]. However, these forecast systems are normally based on predictions featuring a widespread region and a long lead-time. For both precipitation and flood forecasts, the results are not necessarily in line with the real situation and it is difficult to obtain precise results for small local areas, because of the various uncertain factors in the natural climate system, e.g., the complex interactions between hydrology, monsoon, ocean currents, and clouds. Therefore, many studies are currently being conducted with the aim of improving these forecast models. At present, it is still not easy to achieve reliable accuracy for precise regional flood forecasting in a given small area.

Besides the weather and precipitation simulations using forecast models, water-level observation stations use various water-level measuring sensors to implement *in-situ* water-level measurements, which not only provide important observation data for weather forecast systems for further analysis and prediction, but can also be used for providing instant flood warnings. The instant water-level monitoring techniques for precipitation mainly focus on the measurement of the relative height of the water surface. In general, four types of measuring sensors are used for quantitatively determining the water level: Pressure sensors, bubble gauges, float gauges, and non-contact radar gauges. These sensors are used for performing on-site measurements of the water level in rivers, drainage systems, sewers, flood storage ponds, and reservoirs [10–14]. Previously, pressure sensors, bubble gauges, and float gauges were mostly used for this purpose. However, pressure sensors have to be placed at the river bottom or river surroundings, which leads to a relatively high maintenance cost since they can be easily destroyed or buried by floods or the accompanied debris and sands. Float gauges have to be stored in the water-level towers built in the middle of the river, which can cause the problem of high construction costs and difficulties. Bubble gauges use extended measuring tubes to separate the main body from the river. These extended measuring tubes need to be replaced when damaged by flood or buried by debris, which reduces the maintenance cost by well protecting the important main body of the measuring device. However, since the extended tubes still need to be placed inside the river, it can be difficult to maintain a high applicability. Moreover, the extended tubes inside the river require frequent maintenance. On the other hand, non-contact sensors such as ultrasonic gauges or radar gauges [15–20] are expensive themselves and can only be set up where there are structures across the river, which are usually road or railway bridges. This obviously will limit the location, range, and sensor density of the water-level observation. Moreover, such a technique uses the acoustic or optical reflection principles to measure the height of the water surface. Hence, the reliability of the measurement can be easily affected by a number of factors, such as the angle between the radiation source and the water surface, air quality, humidity, water ripples, rain, fog, and the atmospheric media.

Besides the abovementioned precipitation forecast models and direct physical water-level measurements, image-based water-level measurement techniques have been proposed in many recent studies, which employ remote video surveillance images to detect water edges and then convert them to water-level results [21–26], or use continuously filmed images to analyze the surface stream velocity of water [27]. In most image-based water level measurements, the boundary between the datum marks/rulers that are preset in the water and the water surface is considered the water height. After obtaining real-time images of the ruler and the region of interest of the water surface through remote video streaming, we can identify the water surface junction by using image-processing techniques. The water level is then determined by using a “mark to real-world coordinate transfer matrix”, or a preset ruler with absolute height. However, such a technique is limited by the installation location of the marks or rulers and the requirement of high-quality images. The rulers are usually set on the existing piers or embankments—the ones that have been set up in the middle of a river can be easily destroyed, which limits the available choices and extensiveness of the observation locations. In the meantime, in order to perform an image analysis to determine the water level, the visibility or readability of the marks or rulers also need to be maintained. Furthermore, factors such as the camera focal length, camera angles to the rulers, and the relative displacement between the camera and the rulers are all sources of uncertainties during image reading. In addition, good lighting conditions and clear image quality are required for detecting the boundary between the water surface and the ruler. However, in the real outdoor world, the lighting conditions are usually either inadequate or excessive and the key timings for detection are usually within the severe typhoon/thunderstorm period. Therefore, the results may have considerable noise interference, which adds even more uncertainty sources to images in which the water boundaries need to be precisely detected.

Some studies use optical diffusion and reflection principles to implement nonintrusive water-level measurements with external light sources, but some parts of these techniques have to be applied in storage barrels with a low light inference and a high water transparency [28–30]. For example, [29] uses the horizontal diffusion proportional change in light when it shines in transparent water bodies with different water levels. The water level in the tank can be subsequently calculated by using the conversion function. Such optical non-contact measurements are usually adopted when the liquid to be measured is an acid or an alkali or is prone to chemical reactions, in order to avoid the pollution caused by the chemical reactions on the detector surface, or employed in delicately sealed tanks. However, it is difficult to utilize such methods in a real-life outdoor environment to measure the flood water level.

Besides on-site images used for the water-level image analysis, image-based flooding information includes large-scale aerial and remote images from satellites [12,31,32] and synthetic aperture radars (SARs) [33–36], which are normally used for a large-scale geographic analysis on the flood overflow area. However, due to the complex obtaining process, the time delay caused by the large amount of post-processing work, satellite or aerial images are usually used in non-instant applications, such as meteorology, hydrology, and disaster management. Further, it is not easy to obtain precise data of the flood level or the overflow area within a certain small area from aerial photographs. For near real-time flood detection and emergency flood warning [37,38], aerial images cannot effectively observe the near real-time status of floods or obtain accurate flood overflow data, particularly when a specific small area, such as the surroundings of a certain river segment, inner city rivers, urban ditches, flood discharge waterways, and road bridges, needs to be monitored.

The near real-time provision of precise information about flood dynamics from video monitoring is an essential task in disaster management. In this paper, we propose a new surveillance video-based flood monitoring system. Beyond the current flood warning analysis and notification systems, which only rely on precipitation forecasts and water-level sensors, the proposed system is capable of providing near real-time remote surveillance video and automated flood monitoring and warning-level analysis. The key advantage of this system is the introduction of a new video surveillance concept, in which the flood overflow is considered a monitoring object, and the risk level is determined on the basis of the number of preset warning points intruded by the flood object. This method avoids the common needs of the currently used water-level measuring techniques, *i.e.*, suitable locations and structures (e.g., piers or embankments) for setting up the rulers. Moreover, the proposed system does not need high-quality images as required by the currently used image-based water-level measuring techniques for the analysis, nor does it need the cameras to be completely fixed in order to ensure an accurate overlap of the ruler and the region of interest. This technology can improve our monitoring and emergency warning abilities against flood overflow and inundation events, serving as a complement to the currently used quantitative precipitation forecasts and *in-situ* water-level measurements. It is also expected to provide more timely and accurate flood warning information to disaster relief units and the general public, in order to reduce the negative impacts of the flood disasters.

## 2. Image-Based Flood Alarm System

This study focuses on the dynamic detection of floods, and overflow/inundation is considered an intrusion object in the video surveillance image. A surveillance video from a small-scale field of view is used as the input source in order to monitor the water flow and overflow trends in the image. An image segmentation technique is used for removing the surrounding objects, such as buildings and the geographical background, and separating the intrusive objects for a subsequent risk analysis. The image segmentation technique was developed many years ago and is currently widely used in the industrial and medical sectors [39]. For example, it has industrial applications in image-based automatic product quality tests, material defect tests, and package integrity tests. In medical industries, it is used for identifying the locations of tumors or other pathological objects, testing early tumor lesions, measuring the volume of organs, performing image-based diagnoses, and developing treatment programs. In our daily life, this technique is used as the basis of face recognition, environment or traffic monitoring, and robot vision. In recent years, it has also been widely applied in telemetry video analyses. For instance, it can be used for identifying and separating certain objects in the field of geographic information, to obtain information about various environmental objects, such as lands, oceans, cities, forests, and agricultural areas. Traditionally, image segmentation can be roughly divided into four algorithm categories: (a) point-based segmentation; (b) edge detection-based segmentation; (c) region-based segmentation; and (d) hybrid segmentation combining at least two of the above algorithms [40]. In addition to the region-based image segmentation method, some researchers have proposed boundary-based, graph-based, and statistical-based image segmentation methods [41–44]. However, various image properties and interference sources under outdoor weather conditions, such as random changes in the flooded area, reflection from the impurities in air, storms in a bad weather, high level of noise in the images obtained from a heavy rain, and water stains on the camera lenses, can potentially have a negative influence on the accuracy of the traditional image segmentation techniques. For example, background subtraction, threshold, and watershed would not be able to identify the correct boundaries of the flooding objects in an easy manner when the outdoor atmosphere and environment change drastically. Therefore, a region-based image segmentation method and a flood-risk classifier are proposed in this paper, in order to identify the on-site variation of the rivers overflow area and determine the corresponding risk level. Of course, besides monitoring rivers, this method can also be used for flood detection in floodways, ditches, sewers, roads, railways, important areas (e.g., airport runways), or the surroundings of these important facilities. The proposed image-based flood alarm (IFA) system can provide basic information for disaster prevention and warning. This system is connected to outdoor surveillance cameras in the case of high-risk rivers or other hotspots. It automatically estimates the flood risk of the observed area on the basis of the flood area and a risk classification method described in this paper, and then determines whether a prompt initial flood alarm should be given to relevant organizations and staff on the basis of the risk evaluation results.

### 2.1. Structure of the IFA System

**Figure 1.**
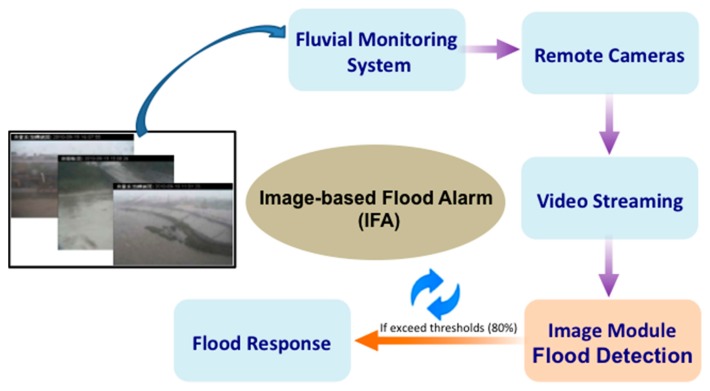
Flowchart of the proposed image-based flood alarm (IFA) system.

Today, cyber surveillance systems have already been widely used in many different fields [45–52]. US National Science Foundation (NSF) first used cyberinfrastructure (CI or Cyber) as a term, which typically is used to refer to modern information technology systems that consists of computing systems, data repositories, sensor instruments, data analysis systems and, visualization environments, and people, all linked by high speed networks to make possible scholarly innovation and discoveries not otherwise possible [53,54]. One of their main applications is long-term, remote surveillance and monitoring, such as safety monitoring, security surveillance, and border surveillance. They are also often used in ecosystem, environment, and underwater observations. Besides real-time videos for remote surveillance, a video surveillance system can record the activities and content changes of all the objects and scenes within the field of view, which can be developed into a recording history database for future use and analysis. The proposed IFA system is an outdoor flood warning system based on surveillance videos and is capable of automatically detecting, evaluating, and reporting flood risks in near real-time. It is an unattended active surveillance and early warning system. The IFA combines on-site real-time video images with a backend image-processing module to conduct a near real-time river overflow and ground inundation analysis. After the image processing module calculates the near real-time water overflow range, the system will automatically provide flood alarms if the overflow range intrudes the preset warning signs. The flowchart of the system is shown in Figure 1, which includes the fluvial monitoring system and the remote cameras. The video streams are transferred to the backend flood detection/image module via the Internet for a flood object analysis and the subsequent flood risk warning. The detection/image module of the image-based IFA system proposed in this paper is composed of two major components: (1) image processing module, which divides the images into foreground (*i.e.*, water stream or flood) and background (*i.e.*, geographical environment, bridges, buildings, and the sky); and (2) flood risk detection module, which evaluates the risk level of the current overflow range and decides whether to send the first risk warning depending on the calculated results. Details of these modules are provided in Sections 2.2 and 2.3.

### 2.2. Image Processing Module

In the application of the IFA system, the inundation or overflow range needs to be actively detected. The flood location and overflow range will then be used for analyzing the current and future flood risks, and eventually provide near real-time flood warning information. This flood information can be transferred to an existing early warning system, or be broadcast to the local public to help them understand the inundation situation and conduct precautionary actions. Real-time videos of river surroundings or urban areas can be easily obtained from the existing remote video surveillance systems. However, various uncertainties in the outdoor environment may affect the video quality. As a result, simple pixel intensity classification methods, e.g., using a certain threshold to distinguish the foreground and the background, are not sufficient to precisely isolate the flooding area. This is due to the fact that with varying weather and time, the color intensities and the shapes of both the foreground and the background are influenced by illumination, fog, rain, and other atmospheric conditions. Sometimes, these visual noises may even appear in a different manner within the same dataset of video streams. For example, the rain and fog distribution in the previous frame is similar but fundamentally different compared to that in the next frame. Therefore, if a certain color or pixel intensity is used for defining the flood range, the definition may easily become ineffective because of the great color variation in the image. However, the region-based segmentation method first selects a certain pixel in the region of interest as the starting point, which is also known as the seed point. When the pixel intensity of the seed point is used as the initial condition, pixels with similar features—based on the preset criteria—are considered to be in the same region. The size of this region increases with the entry of nearby pixels; therefore, this process is also known as region growth. Since this method only requires the preset position coordinates of the seed point and not the range of the pixel intensity, the threshold for segmenting the foreground and the background is more flexible. As a result, it can be employed to perform continuous segmentation for a particular area even when the colors or intensities of the pixels vary in each frame. Therefore, region-based segmentation methods are more suitable for situations where the background environment and the shape of the foreground change over time.

In order to maintain the image consistency for the analysis, river surveillance videos can be obtained from a camera in a fixed position, which usually points at a view that includes both the riverbank and the river course. Besides the position-fixed cameras, in a camera system with pan-tilt-zoom (PTZ) control, the auto-reset function can be used for switching the field of view back to the preset orientation and position when inundation detection is needed, in order to ensure the applicability of the priori seed point location. The seed point only needs to be set once on each camera lens to perform a long-term analysis thereafter. Because of the advantage of a fixed field of view and the normal continuum feature of the flood, region-based segmentation is preferred in this situation. The seeds can serve as the reference of image segmentation for all the subsequent input images once they are predetermined according to the user experience or the real situation.

Based on the above reasons, region-based image segmentation methods are considered more suitable for flood region detection. The main characteristics of region-based image segmentation methods include the following: Firstly, the image segmentation result is related to the seed point location but not in an absolute relationship; *i.e.*, the seed point only needs to be located approximately in the potential flood region. Therefore, precise positioning is not necessary and a relatively high position error tolerance is possible, which ensures that any general staff can easily set up the seed point. Further, because of this feature, the orientation of the camera does not need to be completely fixed, which saves a considerable amount of labor and time cost incurred for calibrating and maintaining the camera position and orientation. Secondly, image segmentation can be done without presetting the pixel intensity range for the foreground and the background. Therefore, all the shape and size variations of the flood over time can be detected and traced.

**Figure 2.**
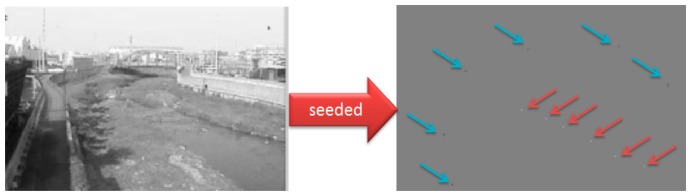
Example of seed point setup. The figure on the left shows the originally observed image, and the figure on the right shows the set positions of seed points, including both the river water part in the foreground and the surrounding environment (*i.e.*, embankment and the sky) in the background. The seed points include two sections: The water region (indicated by red arrows) and the background section to be removed (indicated by the blue arrows).

The objective of region-based image segmentation is to bunch the given seed points with their surrounding pixels to form a meaningful region, such as the water body, the sky, and the embankment. This study adopts the GrowCut method [55] for region segmentation, which is developed from the seeded region growing method. In this method, a cellular automata (CA) algorithm is added to the region growing process to simulate the competition between the foreground and the background during segmentation. In the CA evolution model, each pixel is treated as a cell, which tends to grow outwards and compete with other cells. Similarly, the GrowCut region growth process also starts from the seed pixel and expands outwards, with an attempt to occupy all the pixels in the image. GrowCut has two types of seed points: Foreground and background; each contains at least one pixel as shown in Figure 2. The similar classification also applies for the cellular status, where the foreground and the background regions compete with each other according to the region growth criteria. The GrowCut region growth criteria are also called the local transition function. This function is used for predicting how the status of the current cell (pixel) will change after interacting with nearby cells. The effect of the function is the same as that of the traditional region growth criteria and both are criteria for determining whether a cell or a pixel should be treated as the foreground or the background. On the other hand, the local transition function is different from the traditional region growth criteria in that the status of pixels already included in one of the regions may possibly be changed by pixels in the neighboring region during the growth process, which results in long-lasting back and forth changes in the shape and size of the foreground and background regions. Therefore, the region growth of CA can proceed in both directions. The growth/competition does not stop until all the criteria are satisfied. This method can effectively deal with the blurry and glow scattering regions created by the stains on the lens, which enables the foreground region to grow across the stain boundaries through competition in order to identify a more complete overflow region.

The pseudo code of the GrowCut CA evolution pattern is described as follows:
% For each cell in an imagefor V*_p_* ∈ *p*  % Copy previous state  
$l_{p}^{t+1}=l_{p}^{t}$;  
$\theta_{p}^{t+1}=\theta_{p}^{t}$;  % Neighbors try to attack current cell  for V*_q_* ∈ *N*(*p*)   if 
$g\left({\left\|{\vec{C}}_{p}-{\vec{C}}_{q}\right\|}_{2}\right)\cdot \theta_{q}^{t}>\theta_{p}^{t}$    
$l_{p}^{t+1}=l_{p}^{t}$    
$\theta_{p}^{t+1}=g\left({\left\|{\vec{C}}_{p}-{\vec{C}}_{q}\right\|}_{2}\right)\cdot \theta_{q}^{t}$   end if  end forend forwhere *P* represents the pixels in the entire image, which can have two or more dimensions; *p* denotes the currently executed pixel; q indicates the remaining pixels to be processed; L refers to the label of the current pixel, representing foreground, background, or not processed (zero); **θ** denotes the intensity of the current pixel, with an initial value of zero; *N*(*p*) represents the neighboring pixels of the current pixel; and *C_p_* and *C_q_* indicate the eigenvectors of this pixel. The initial *C_p_* and *C_q_* values are composed of the intensities in the three channels of its RGB color. In order to simplify the calculation and speed up the image segmentation to achieve the near real-time requirement of the system, the JPEG images used in this study are converted to grayscale prior to the segmentation process. In particular, the original JPEG image with the RGB color model is converted to the HSV color model. Then, only the *V* value is taken as the grayscale intensity. Thus, the original three-channel processing is simplified into a single channel.

**Figure 3.**
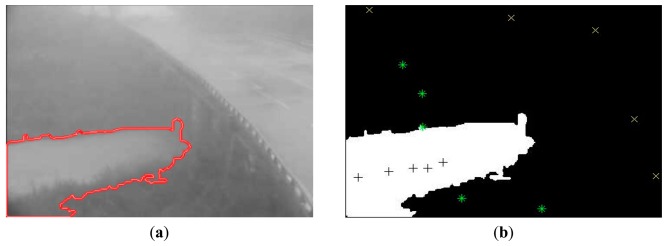
Example of flood region detection. Figure (**a**) shows the detection result for the river water region, indicated by the red boundary. In Figure (**b**), the white area denotes the flood region, foreground seed points are represented by black plus signs (+), background seed points are represented by yellow “x” symbols, and the user preset warning points are represented by green asterisk symbols (*). In this example, none of the preset warning points are intruded by water; therefore, the risk is 0%.

Foreground and background labels are first allocated to the seed points of the input images following the traditional region growth method. Then, the abovementioned CA algorithm collects pixels that satisfy the criteria via a region competition, in order to form a larger independent region. As a result, the foreground and the background compete with each other and either grow or decline. This segmentation process proceeds until no more pixels can be labeled. In this scheme, the traditional region growth method is employed to allocate labels for a few seed point pixels before the adoption of the CA algorithm for region competing growth. Hence, it is called the GrowCut method, which indicates that it takes advantage of both the region growth method and the CA segmentation competition. As shown in Figure 3b, the foreground, *i.e.*, the river body that we want to detect, has its seed points represented by black plus signs, while the background, *i.e.*, the environment objects to be removed, such as the land, road bridges, and the sky, are denoted by yellow “x” marks. The preset flood warning points are represented by green “*” marks. The eventually identified flood region based on the above algorithm is used for determining the flood risk together with these warning points.

### 2.3. Flood Risk Detection

Flood risk detection and warning are included in the image-processing module to detect the risk level of the flood region and determine whether a warning should be generated on the basis of the risk level. Assuming that the flood is an invasion object and the entire field of view is the our surveillance view, the overlap proportion of the detected overflow region and the preset warning points are used as the standards of risk estimation. For example, if there are five preset warning points and four of them are invaded, or included in the overflow region, then the risk is 80%. Figure 3 shows a real example of the IFA system detecting a floodwater region. Figure 3a shows the detected water region, which is indicated by the red boundary; in Figure 3b, the white area represents the identified water region, the foreground seed points are represented by black plus signs (+), background seed points are represented by yellow “x” signs, and the user preset warning points are represented by green asterisk symbols (*). The flood risk detection and the warning thresholds are calculated as follows:

(a) *A_j_* ∪ *R_water_*, where *A_j_* denotes an alarm point (*j* = 1,2,…,*k*) and *R_water_* represents the water region of interest.
(b) Risk =[*A_j_* ∪ *R_water_*/*k*] ×(%)
(c) If Risk > 80%, warning.

where *A_j_* denotes the priori preset warning position point, which is set by technicians from the responsible unit according to specific views in different locations; *j* = 1:*k* represents the number of warning points, with the maximum value of k suggested to be 5 to 10. *R_water_* refers to the identified overflow region in the image calculated by the proposed algorithm. The overflow region is shown as the white area in Figure 3b, while the background is shown in black. The portion of *A_j_* included in *R_water_* is the current flood risk level. In other words, Risk is the percentage of alarm points overlapping with the water region. When the risk level is above 80%, the first warning should be sent. These warning can be superimposed on the Taiwan topographic map of the hydrological monitoring network. As shown in Figure 6 (Section 3. Test Case), the surveillance video and location information of the place with an extra high-risk level can directly pop up on the webpage. In the meantime, these locations are displayed in a striking red color on the map, in order for the monitoring staff to easily see the overflow situations in each region. This information can also be sent to the general public or other relevant people through cellphone apps, texts, or emails.

### 2.4. Influence of Atmospheric Conditions

At this point, we have implemented the operational tools within a cyberinfrastructure platform, the image-based flood alarm (IFA) system, to monitoring the near real-time flooding level on the video streaming of remote cameras. What remains is the influence of atmospheric conditions that affect the image processing results. Severe atmospheric conditions exert complex visual effects for outdoor imaging, such as fog and raindrop stains. For the fog, the rainstorm involves suspended particles, mist, raindrops, raindrop streaks, and heavy rain spray; they add a non-uniform fuzzy mask between the scene and the camera render images extremely unclear. On the other hand, raindrop stains tend to adhere to the lens of cameras that refracts and reflects the light to generating shape and intensity changes in recorded images. These conditions considerably weaken the image quality of cameras and the outcomes of subsequent image processing. In proposed system, GrowCut exhibited superior resistance to rain stains, but yielded segmentation failure in the period of dense fog and the nighttime.

## 3. Test Case

**Figure 4.**
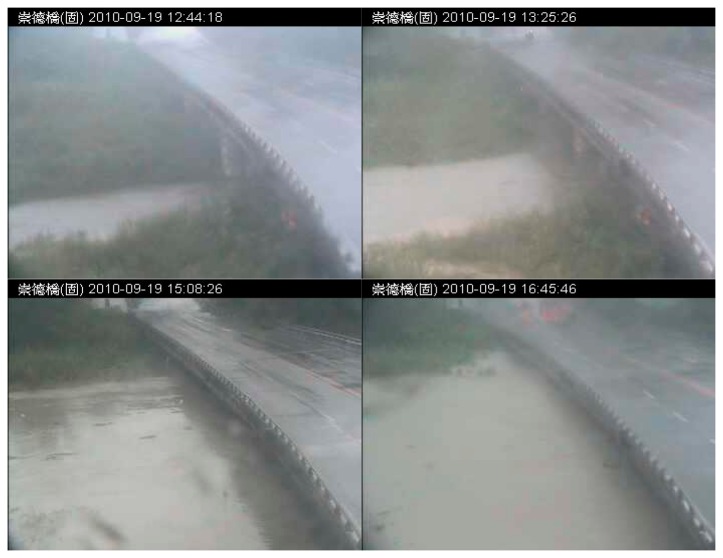
Video screenshots from the water-level detection test. This figure has four best-quality images showing representative overflow changes. From top-left to bottom-right, the acquisition time of the four images is 12:44, 13:25, 15:08, and 16:45, respectively. The content of the experiment is the real video of Typhoon Fanapi taken on 19 September 2010, from 12:00 to 18:00 in the afternoon. The experimental images were captured every 1 min during this period, with a total of 350 images.

The proposed flood detection system was tested using a real outdoor surveillance video case. The proposed video processing module and flood risk detection method were adopted in this test case to identify the flooding area and analyze the flood risk. The remote surveillance video used in this experiment was taken from 12:11 to 5:50 p.m. on 19 September 2010, when Typhoon Fanapi passed by. It was the peak rainfall period during Typhoon Fanapi, so the video recorded the sudden flood overflow caused by the heavy rainfall within a short time, at the Chongde Bridge section of the Erhjen River basin in the Tianliao district of Kaohsiung. The outdoor surveillance video system transferred the on-site real-time video back to the host computer via the Internet, and provided online monitoring, video archiving, and automatic video analysis for the water resource departments. On the host computer, screenshots were taken at 1-min intervals from all the surveillance video streams returned from the on-site cameras for a further analysis. The 1-min interval was set because the flood area usually does not change dramatically within 1 min and thus, the detection can still be considered a near real-time flood overflow detection with the 1-min delay. Moreover, the computational load and storage costs could be significantly reduced in this way. This real-life case covered a time period of about 6 h. A total of 350 images were therefore extracted for the experiment, and each of these JPEG images has a resolution of 352 × 288 pixels. Some of the tested images are shown in Figure 4, including those of the piers and the deck of the Chongde Bridge on the right side and the Erhjen River and its two banks in the center. Starting from around noon, the river water level quickly increased because of the heavy rainfall. The water level reached its peak between 3:00 p.m. and 4:00 p.m., which was close to the road on the top of the bridge.

The proposed image-processing module can continuously perform segmentation for the input flood video stream by using the foreground and background seed points, and keep complete records of the flood region variations over time. The detected results for the flood objects will subsequently be used for the flood risk analysis. It has been experimentally proven that there is no stringent requirement on an accurate selection of seed points, which is in good agreement with the previous theoretical assumption. For example, the foreground seed points only need to be placed in the possible flooding area instead of the background. Therefore, the same flood detection result can be achieved without a 100% precise setting of seed point locations. The same is true for the background seed points. However, note that the background seed points have to be placed in locations that are unlikely to be reached by the flood when the water level increases. The algorithm used in this study only treats the seed points as the starting point of the initial image labeling and the final segmentation result after the iterative CA competition process is not restricted by the location of initial seed point but depends on the overall properties of the foreground and the background instead. However, the outward growth of the foreground may be slightly weakened if the background seed points are too close to the foreground seed points in practical cases, which will result in a withdrawal of the foreground boundary near the background seed points. Therefore, the use of prior tests or experience to set up the seed points will be helpful in obtaining a better and more complete segmentation of the flood area.

**Figure 5.**
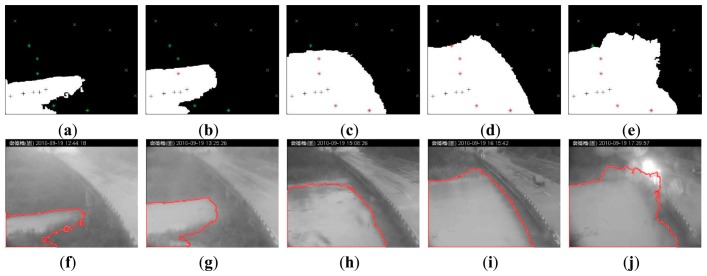
Test results of a real-life case. Figures (**a**) to (**e**) show the foreground seed points (+), the background seed points (x), and the preset warning points in green (*) marks in the processed binary images. In these binary images, the white segments indicate the detected water area, while the black segments show the background objects (e.g., ground, the sky, and bridge) to be deleted. Figures (**f**) to (**j**) show the flooding area detected from the input video, indicated by the red boundary.

All the real-life images were processed by the image-processing module to detect the flood object area. The flood detection results of this practical case are shown in Figure 5. In Figure 5, the grayscale images in the left column are transformed from the input images, with red boundaries indicating the identified flood objects. Images in the right column are the corresponding flood intrusion analysis results. In the right-hand side images, white regions represent the flood objects, equivalent to the flood areas inside the red boundaries in the left-hand side image, and the black parts are background areas. In addition, foreground seed points are represented by black (+) marks, background seed points are represented by yellow (x) marks, and preset warning points are represented by green (*) signs. The preset warning points invaded by the flood objects are represented by red (*) signs. Figure 5 only shows the images at five different time points, from Figure 5a–e, 12:44, 13:25, 15:08, 16:15, and 17:39, respectively. Using the flood risk detection method introduced in Section 2.3, we can estimate the risk level by using the portion of preset warning points covered by the flood objects. In addition, the preset warning threshold of the system for the first warning is 80%, in order to avoid unnecessary repeated warnings. Figure 5 shows the detection results and flood risk levels at five different time points. In Figure 5g, only one warning point is covered by flood, so the flood risk is 10%. However, in Figure 5h,j, four of the five preset warning points are covered by flood, so the flood risk is 80% at these times. The flood risk levels in Figure 5f–j at five time points are 0%, 20%, 80%, 100%, and 80%, respectively. A real-life flood event that resulted from a typhoon was used in this test for system validation. All of the surveillance images with an interval of 1 min were processed by the IFA system to detect the water level and analyze the flood risk. Each image at a particular time point was segmented to identify the flood object. The flood risk was then calculated on the basis of the number of warning points being invaded by the flood object.

**Figure 6.**
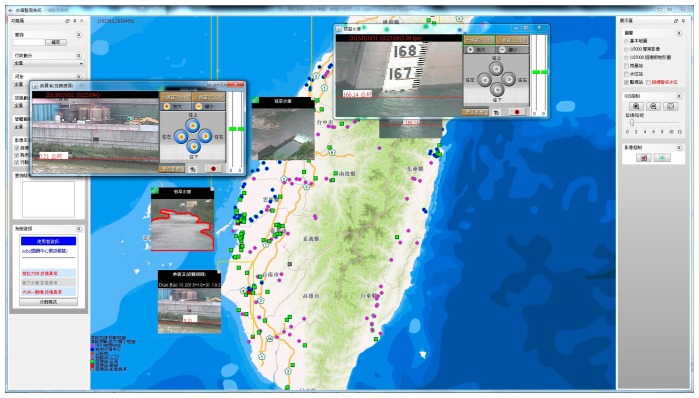
Online user interface. Appearance of the hydrological monitoring and flood risk warning system in operation. If any monitoring station reaches a risk level of 80%, the video from the on-site camera directly pops up on the interface, with a red outline indicating the near real-time detected flooding area.

With the automated image processing method, a large number of remote surveillance videos at various locations can be simultaneously monitored for a flood warning service. This can be combined with river monitoring images and city traffic surveillance images to perform large-scale near real-time flood detection and obtain information about the overflow area. Warnings are subsequently sent to the relevant staff on duty and online disaster prevention monitoring systems if the risk levels of the rivers or the other sections are higher than the threshold values. An automated, quick, and easy flood warning service can be provided to replace the manned monitoring. Figure 6 shows a combination of the proposed image processing system and the existing flood monitoring screen. Locations with high-risk levels automatically show up on the flood monitoring platform. Surveillance videos and identified flood events at high-risk locations automatically pop up on the interface or get circulated to specific staff in other ways.

## 4. Conclusions

The main purpose of this study was to improve the traditional surveillance image-based water level analysis and its relevant techniques for the detection of floods, overflow, and inundation. Traditional image-based techniques have been restricted by a number of disadvantages, such as the location choice of the ruler setup, the lack of a large field of view covering sufficient geographic information, the precise calibration between the camera and the ruler, the maintenance requirement for maintaining the fixed calibration status, and the scale conversion from the image size to the real-world size. This study proposes the use of the object intrusion principle to monitor a flood or inundation event and analyze the corresponding risk level. It has been proven by using a practical test case that the proposed methodology can correctly detect the changes in flood overflow and estimate the near real-time flood risk levels on the basis of the degree of flood intrusion.

Traditionally, when a remote surveillance video is used for detecting the water level, the primary principle is to obtain the overlapped position of the water surface and the height on the ruler [23,26] or the relative distance between the water surface and the graphical ruler [21,25]. The image is first processed to get the water surface position. Then, the real-world water surface height can be obtained using the relative geometric relationship between the water surface and the ruler. This method can deal with a small field of view and is capable of getting data for the water surface height, as stated above; however, it still has many restrictions. The intrusion monitoring method proposed in this study has been playing a vital role in the world of video surveillance. Intrusion monitoring can be used for monitoring intrusive activities in a given space [56–58]. Therefore, it can be used for directly monitoring flooding events or inundation regions, without the need of converting the image water surface height to the real-world scale.

As expected, the practical test case revealed that the outdoor image-based application is influenced by weather conditions and atmospheric visibilities. In particular, during heavy rainfall or night time, camera lenses that rely only on visible light are more easily affected by atmospheric visibility, which is consistent with our previous study [37]. In addition, one limitation of the proposed method is that it cannot directly obtain the height data for the absolute water level but can determine the scale of the flood on the basis of the preset warning points in the image. Therefore, it is not suitable for a quantitative recording of the water level. Moreover, the setup of the warning points in the surveillance image depends on the decision of the professional staff. Different positions of warning points will slightly affect the consequent calculations of the risk levels. However, the advantage of this system is that it avoids the complex work required by the previous image-based water-level estimation techniques. For example, it does not require the preset of the ruler for measuring the water level or the use of an existing bridge pier or riverbank as the carrier for the ruler. As a result, it is more flexible in location selection for flood detection and does not need the post-processing work to convert the water surface height from the image scale to the real-world scale. The proposed flood intrusion detection system has a considerably lower image quality requirement than the traditional boundary identification between the water surface and the ruler, and has a relatively high tolerance for the noise caused by bad weather conditions. It also has a lower maintenance requirement for calibrating and maintaining the region-of-interest position of the camera. Since there is no need to limit the field of view of the camera on the region of interest of the ruler, the camera can cover a larger range of *in-situ* visible geographic information for visual verification. Eventually all the measured data can be easily confirmed by visual verification. All these advantages make up for the shortcoming of obtaining only the water level height information from previous image-based methods. Therefore, this proposed method has a relatively high robustness in outdoor flood detection and warning applications.

This study proves that the video surveillance theory can be used for providing an unattended flood detection and warning service. In addition, this automated detection system can perform a near real-time analysis on a large number of remote surveillance videos at the same time and provide an automated, quick, and convenient flood warning service. Similarly, it can be widely used in urban inundation detection. Combined with the currently existing traffic and security surveillance videos, it can be employed to automatically monitor the overflow of a city's inner river or the inundation of streets, which will help citizens stay away from the inundation regions and reduce the hazards brought about by urban inundation.
